# Urban digital economy, environmental pollution, and resident’s health–empirical evidence from China

**DOI:** 10.3389/fpubh.2023.1238670

**Published:** 2023-12-07

**Authors:** Chen Zhu, Zekai Wang, Bin Sun, Yuanyuan Yue

**Affiliations:** ^1^Business School, Yangzhou University, Yangzhou, China; ^2^School of Economics, Dongbei University of Finance and Economics, Dalian, China; ^3^School of Marxism, Dongbei University of Finance and Economics, Dalian, China

**Keywords:** digital economy, residents health, environmental pollution, 2SRI method, *U*-test

## Abstract

In light of China’s rapid advancement in the digital economy and the implementation of the “Healthy China” initiative, it is crucial to assess the impact of the digital economy on residents’ health. This study analyzes data from the 2012, 2014, and 2016 China Labor Force Dynamics Survey (CLDS) to evaluate the health of residents using both subjective and objective criteria. Furthermore, it calculates the digital economy development index for Chinese cities and investigates its influence on the subjective and objective health of residents, along with the underlying mechanisms. The empirical results reveal a U-shaped pattern in the effect of the digital economy on health levels, initially detrimental but subsequently beneficial. The analysis of mechanisms shows that the digital economy’s development initially increases and then decreases environmental pollution, impacting health through environmental changes. Additionally, the study finds variations in this impact based on age and urban–rural differences, with more pronounced effects on rural and older adult populations, who also experience the U-shaped curve’s turning point more rapidly. These findings highlight the necessity of advancing digital economy infrastructure to positively influence environmental quality and improve public health. The study emphasizes the urgent need for policymakers to invest in digital infrastructure to foster a sustainable and healthy future. This requires a holistic approach to development, focusing on both urban and rural areas, to promote inclusive growth and reduce the digital divide.

## Graphical abstract

GRAPHICAL ABSTRACT

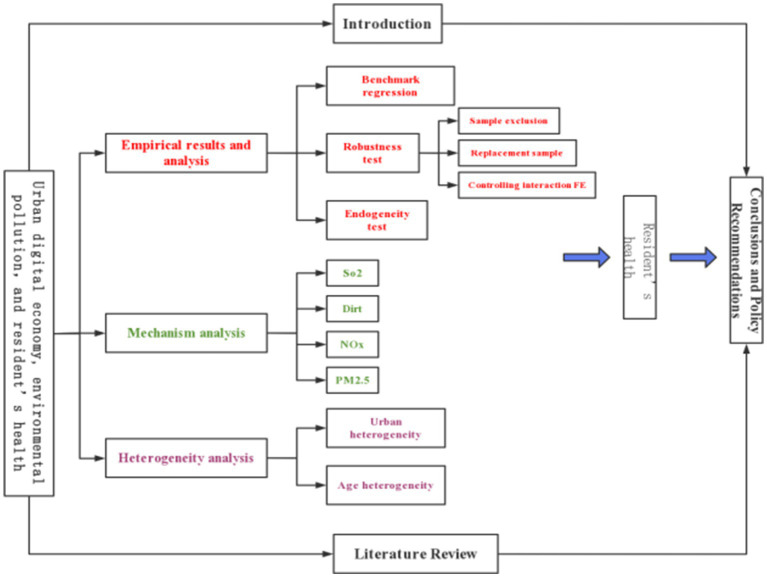

## 1 Introduction

Amidst rapid global economic development, population aging has emerged as a significant concern, drawing increased international attention to health issues. Health, as a vital component of human capital, is essential for ongoing individual development and long-term national prosperity. The World Health Organization’s Global Health Strategy recognizes health as central to the global development agenda. Additionally, World Bank data indicate that national healthcare system investments substantially enhance life expectancy and quality of life, emphasizing health’s role in fostering social progress. Therefore, improving residents’ health status is a key indicator of societal development and a direct reflection of social progress.

The digital economy, characterized by digital knowledge and information as its primary production elements and modern information networks as its main carriers, has become a new economic development model. Propelled by the internet revolution, the digital economy’s rapid growth is a global trend. The International Data Corporation (IDC) reports that the global digital economy reached $11.5 trillion in 2021, accounting for over 15% of the global GDP, and is projected to increase to $16.5 trillion by 2022. This growth signifies the digital economy’s major role in global economic expansion. The 2022 China Digital Economy Development Report reveals that China’s digital economy amounted to 45.5 trillion yuan in 2021, comprising 39.8% of the GDP, and has become a significant driver of both China’s and global economic growth. Similar trends are observed in other countries and regions, making the digital economy a vital part of the global economic structure.

While factors such as education (1), subjective social status (2, 3), social mobility (4), and income (5) significantly impact resident health, research on the digital economy’s influence in this area is relatively scarce. This paper, using China as a case study, explores the potential relationship between the rapid development of the digital economy and the improvement of resident health levels. It raises a global question: Is there a universal correlation between the digital economy’s development and resident health improvement? If so, what mechanisms are involved? Focusing on environmental pollution, this study examines the digital economy’s impact on the environment, a known factor affecting resident health (6), and analyzes its potential effects on health levels, aiming to provide a new perspective on this global issue. The study uses data from the 2012, 2014, and 2016 China Labor-force Dynamics Survey (CLDS) and evaluates resident health levels from both subjective and objective aspects, combining this with the city-level digital economy index to investigate its impact on residents’ health.

The study’s contributions are multifaceted: It uses CLDS data to measure individual health using subjective and objective indicators, applies the entropy method to calculate the city-level digital economy development index, and analyzes the digital economy’s spatiotemporal evolution. It explores the digital economy’s impact on individual health, revealing a “U”-shaped pattern that initially suppresses and then enhances resident health. The study also confirms the role of pollution mitigation in the digital economy’s impact on health, finding an inverted “U”-shaped influence of the digital economy on environmental pollution, which in turn affects resident health. Additionally, it examines the impact of the digital economy on various population groups, analyzing age and urban–rural differences in health outcomes, providing a comprehensive view of the digital economy’s effect on resident health.

The paper is organized as follows: Section 2 reviews the relevant literature on the digital economy, environmental pollution, and resident health; Section 3 outlines the research methodology and data; Section 4 discusses and analyzes the empirical findings; Section 5 delves into the impact mechanisms and heterogeneity; Section 6 concludes with policy recommendations.

## 2 Literature review

In recent years, the digital economy’s rapid global expansion has garnered increasing scholarly attention. As this economy is still in a nascent stage, much of the existing research concentrates on its association with economic growth. This encompasses a range of topics, including digital inclusive finance (8), high-quality economic development (9), regional innovation (7), and industrial transformation (10). In light of China’s swift advancement in the digital economy and the strategic initiative of “Healthy China, “this study seeks to explore the impact of the digital economy on residents’ health and identify the primary mechanisms behind this influence. Specifically, it examines the urban digital economy’s effect on residents’ health and delves into the mechanism of this impact, particularly focusing on environmental pollution. The following sections will offer a comprehensive review of pertinent literature.

A primary consideration is the body of literature addressing the digital economy’s impact on environmental quality. With escalating environmental challenges globally, scholars have begun to scrutinize the environmental repercussions of digital economy growth. This area of study can be categorized into three principal perspectives.

The first perspective posits that the digital economy’s development can markedly improve environmental quality. Several scholars contend that the digital economy has the potential to decrease energy consumption and environmental pollution. This can be achieved through methods including the promotion of industrial agglomeration and the facilitation of specialized labor division, promoting circular economy (11), improving technology innovation efficiency (11), and improving green total factor productivity. These factors promote the development of low-pollution and low-energy industries, which reduces the intensity of pollution emissions (12, 13). Others have analyzed the role of enterprise digitalization and information and communication technology (ICT) in changing the energy structure. They contend that the use of renewable and clean energy by enterprises can reduce carbon dioxide emissions and air pollution, such as haze, thus enhancing environmental quality (14–17). Meanwhile, some scholars have also found that digital transformation of enterprises can promote innovation through channels such as knowledge flow, technical talent, R&D investment, and innovation awareness (11, 18), thereby helping to reduce pollution (19). Furthermore, the development of ICT has led to the promotion of smart cities as a new model of digital urban development by governments. Numerous studies have confirmed that the construction of smart cities (SCC) can alleviate environmental pollution caused by urbanization (13, 20–23).

Conversely, the second perspective maintains that the digital economy’s development could aggravate environmental degradation. Research indicates that the extensive use of modern Information and Communication Technologies (ICT) like Artificial Intelligence (AI), Cyber-Physical Systems (CPS), and the Internet of Things (IoT) has notably escalated energy consumption and carbon emissions. Consequently, the advancement of the digital economy may result in heightened pollutant emissions, thus posing a greater environmental threat (24–26).

The third perspective proposes a non-linear environmental impact of the digital economy, suggesting that the relationship between the digital economy and environmental pollution might exhibit either a “U” or an inverted “U” shape. For example, Ahmadova et al. (27) identified an inverted “U” correlation between digitalization and environmental performance. Their findings indicate that, in a country’s early digital economy stages, digitization can enhance energy efficiency, resource management, and environmental performance. Yet, beyond a certain threshold, excessive digitization may lead to a “rebound effect, “characterized by increased resource consumption and pollution. In a similar vein, Yang et al. (28) observed that digital technology development can mitigate haze pollution, establishing an inverted “U” relationship between the two. According to their research, the impact of the digital economy on haze pollution initially decreases but eventually increases as digital technology continues to evolve. Similarly, Zheng et al. (29) found in a study using Tencent’s digital index that the impact of urban digitization on carbon emissions in China follows an inverted U-shaped curve. Furthermore, Cheng et al. (30) discovered that the digital economy influences carbon emission intensity by optimizing industrial structures and fostering scientific and technological innovation. Their study reveals a “U”-shaped relationship between these factors, accompanied by non-linear spatial spillover effects.

The second segment of the literature review delves into the impact of environmental pollution on residents’ health. Research in this domain predominantly employs Grossman’s (6) health production function, which posits that health is a commodity generated through various investments such as lifestyle, living environment, education, personal income, and healthcare services. The living environment, in particular, is closely associated with health, as its quality profoundly influences residents’ well-being. Numerous studies have substantiated the effects of environmental pollution on health. For instance, Tan et al. (31) examined the influence of air pollution on the health-related quality of life among older adults, revealing that prolonged exposure to PM2.5, PM10, and SO2 heightened experiences of pain, discomfort, anxiety, and depression. They observed that a higher socioeconomic status exacerbated the detrimental impact of air pollution on health-related quality of life. Similarly, Yang and Liu (32) identified a significant adverse effect of air pollution on residents’ self-assessed health. Yang (33) investigated both urban and rural residents’ perceptions of environmental pollution and its health risks, discovering that air, garbage, and noise pollution adversely affected the health of urban dwellers but had a less pronounced impact on rural residents. Zhong et al. (34) explored the relationship between economic growth objectives and residents’ health, finding that the pursuit of these targets often leads to increased PM2.5 levels and industrial solid waste emissions, posing threats to public health. Additionally, studies such as Currie (35) have investigated health inequalities resulting from environmental pollution, noting that individuals with lower socioeconomic status face heightened health risks due to greater exposure to environmental pollutants. These findings collectively indicate that environmental pollution is a critical factor contributing to health disparities.

Conversely, enhancing environmental quality, particularly air quality in residential areas, can positively impact residents’ health. For instance, Zhao et al. (36) discovered that air quality improvements have prevented premature deaths and reduced incidences of cardiopulmonary, lung, and respiratory diseases linked to air pollution exposure. Consequently, regional initiatives to combat air pollution yield substantial environmental, health, and economic benefits. Additionally, Wang et al. (37) investigated the health benefits of policies targeting SO2 air pollution. They found that command-and-control environmental regulations, exemplified by “two control zones” (TCZs), effectively diminish air pollution-related diseases. This reduction is achieved by lowering industrial SO2 emissions, reducing industrial smoke emissions, and promoting increased physical activity among individuals. Liu et al. (38) assessed the impact of China’s clean air policies on resident health. They demonstrated that the implementation of stricter clean air policies can significantly reduce PM2.5 concentrations and the population exposed to PM2.5, thereby decreasing the number of deaths caused by PM2.5 exposure.

The final part of the literature review focuses on the literature examining the impact of digital economy development on public health. While there is limited literature in the academic community directly investigating the impact of the digital economy on residents’ health, some studies explore its indirect effects, which can be valuable references for this paper. Among these studies, He et al.’s (39) research closely aligns with our paper, they examine the health effects of urban digital economy development at a macro level and find that the digital economy can reduce residents’ mortality rates by linearly reducing environmental pollution levels. However, it is important to note that they has not yet been conducted on the heterogeneity of digital economy development’s impact on residents’ health with respect to different social and demographic characteristics. Furthermore, within the realm of public health, numerous scholars have explored the impact of the digital economy on various aspects of public health management, revealing noteworthy insights. McMullan (40), Sims et al. (41), and Odone et al. (42) argue that the application of digital technology in modern healthcare, education, and technology effectively enhances public health by improving healthcare services and accessibility. Additionally, research conducted by Starr (43) highlights the positive influence of the digital economy on the government’s governance capacity, they found digital economy promotes the establishment of a digital government, thereby enhancing the government’s ability to respond to public health events, improve performance, and optimize the efficiency of providing public health services. Moreover, scholars such as Wang and Xu (44), examining the income mechanism, have found that digitalization can positively impact public health by reducing income inequality, which in turn contributes to improved health outcomes. However, it is worth noting that as digitization deepens, some scholars caution against potential adverse health effects arising from the inappropriate use of the Internet. For instance, Sami et al. (45) found that excessive Internet use among adolescents can lead to sleep problems, potentially leading to depression and negatively impacting physical and mental health. Similarly, Bozkurt et al. (46) warn against the detrimental consequences of excessive Internet use, which may reduce physical activity and contribute to obesity problems. These contrasting viewpoints illustrate the multifaceted relationship between the digital economy and public health, underscoring the need for further research and cautious consideration of potential health implications in the context of technological advancements.

The review of existing literature establishes a crucial theoretical base and offers empirical references for examining the interplay between the digital economy, environmental pollution, and residents’ health in this study. However, our analysis also reveals certain limitations in current research, highlighting areas ripe for further investigation: (a) Many previous studies have predominantly focused on the relationship between two of these factors, with less attention given to exploring the interconnections among all three simultaneously. (b) The majority of research has evaluated the digital economy’s impact on regional population health at a macro-level, such as provinces or cities, leaving a gap in detailed analysis at the individual, micro-level. (c) In light of China’s significant regional economic disparities and aging population trend, there is a notable absence of heterogeneous analysis of health status across different demographic groups. These groups might exhibit considerable variations in health status and its determinants. To bridge these research gaps, this paper employs a mediation effect model to scrutinize how urban digital economy development levels affect residents’ subjective and objective health. We delve into the mechanisms linking the digital economy and residents’ health through the lens of environmental pollution, aiming to provide a thorough understanding of the intricate relationships among these elements.

## 3 Model setting, data sources and study design

### 3.1 Model setting

#### 3.1.1 Econometrics model

This paper examines the impact of digital economy development on the health of Chinese residents from a micro perspective, focusing on the effects of environmental pollution.

To this end, the health of residents is divided into two dimensions: subjective health and objective health, based on the characteristics of the questionnaire data. The Ordered Probit model is used to analyze the self-rated health data from the questionnaire, which is an ordered discrete variable:


(1) $$Shealth_{ijt}=\alpha_{0}+\alpha_{1}DEI_{j,t-1}+\alpha_{2}DEI^{2}{}_{j,t-1}+\alpha_{3}C+\sigma_{j}+\tau_{t}+\varepsilon_{ijt}$$

The explained variable “$Shealth_{ijt}$” represents the self-evaluation health of individual i of city j in year t, which is a five-level categorical variable. The explanatory variables “$DEI_{j,t-1}$” are the urban digital economy development index of city j in the previous year (t-1), while “${\mathrm{DEI}}^{2}{}_{j,t-1}$” represents the square term of the digital economy development index. “C” denotes the set of control variables that affect the subjective health of residents. “$\sigma_{j}$” and “$\tau_{t}$” are dummy variables for city j and year t, respectively, used to control for area and time effects. The random disturbance term is represented by “$\varepsilon_{ijt}$”.

Additionally, this paper uses “the need for hospitalization in the past year” as an objective health measure and sets up the following Probit model:


(2) $$Ohealth_{ijt}=\beta_{0}+\beta_{1}DEI_{j,t-1}+\beta_{2}DEI^{2}{}_{j,t-1}+\gamma C+\sigma_{j}+\tau_{t}+\varepsilon_{ijt}$$

Where the explanatory variable ${\mathrm{Ohealth}}_{\mathrm{ijt}}$ denotes the dichotomous variable of objective health of individual i in the city j at the year t. The meanings of other variables are the same as in equation (1).

The regression equation examines the effect of the digital economy on the subjective health of residents by examining the coefficients $\alpha_{1}$ and $\alpha_{2}$, and the effect of the digital economy on the objective health of residents by examining the coefficients $\beta_{1}$ and $\beta_{1}$. If the primary term coefficient is significant but the secondary term is not significant then it indicates that the effect of digital economy on residents’ healthy is linear, and if both the primary term coefficient and the secondary term coefficient are significant then it indicates that the effect of digital economy on residents’ health is non-linear.

#### 3.1.2 U-shape test model

As the impact of the digital economy on resident health may present a nonlinear U-shaped relationship, we referred to the U-test method by Lind and Mehlum (47) to properly test for a U-shaped relationship in our regression model.

In the past econometric literature, many articles attempted to identify nonlinear relationships through regression analysis. However, Lind and Mehlum (47) pointed out that these articles almost never used proper methods when testing for the existence of U-shaped relationships. The common practice for testing a U-shaped relationship should be to check two necessary conditions: whether the sign of the second derivative is correct and whether the extreme value is within the range of the data. However, most studies claiming to have found a U-shaped relationship typically only report the results of the former, and if the estimated extreme value is too close to the end of the data range, then this finding can actually be misleading. To this end, Lind and Mehlum proposed a three-step method for U-test to ensure the robustness of identifying the “U” shaped relationship: (1) the estimated coefficients of the core explanatory variable’s first and second terms in the regression results are significantly opposite in sign; (2) the slopes at the left and right endpoints of the core explanatory variable’s range have opposite signs (Kmin*Kmax<0); (3) the turning point of the curve should be within the range of the core explanatory variable’s values. By using the *U*-test method, we can overcome the issue in past research where the determination of a U-shaped relationship was based solely on the opposing signs of the first and second term coefficients in the regression analysis, while neglecting the possibility that the turning point may fall outside the data range, leading to an actual linear relationship between the variables.

### 3.2 Data sources

The micro-individual characteristics data utilized in this paper are derived from the China Labor-force Dynamics Survey (CLDS) individual and household questionnaires from 2012, 2014, and 2016. The CLDS survey comprehensively covers various aspects of China’s labor force, including education, employment, migration, health, social participation, economic activities, grassroots organizations. As a large-scale interdisciplinary tracking survey, it offers a wealth of data for research purposes.

The CLDS sample encompasses 29 provinces, cities (excluding Hong Kong, Macao, Taiwan, Tibet, and Hainan), and municipalities directly under the central government. For each survey year, the sample consists of 401 villages, 14,214 households, and 23,594 individuals. The survey employs a probability sampling methodology that is multi-stage, multi-level, and proportional to the size of the labor force. Notably, the CLDS is the first in China to adopt the sample rotation tracking method, which effectively adapts to China’s rapidly changing environment and accommodates the characteristics of cross-sectional surveys. The sample is representative of the entire country, including the Eastern, Central, and Western regions, as well as Guangdong Province and the Pearl River Delta. For the study’s purpose, a total of 53,904 valid micro samples were obtained after excluding cases with missing data.

Additionally, the macro-city data were mainly obtained from various sources, such as the China Urban Statistical Yearbook, the “Digital Inclusive Finance Index” compiled by the Digital Finance Research Center of Peking University and Ant Gold Service Group, and the “Compilation of China’s Labor Marketization Index” report published by the group of the Labor Market Research Center of the National Institute of Development and Strategy of Renmin University of China.

### 3.3 Variable definition and measurement

The explanatory variable in this paper is the health of the residents. While traditional studies have mainly used objective indicators such as illness and hospitalization to measure health, recent research recognizes the importance of incorporating subjective health status, or subjective health. Subjective health refers to an individual’s evaluation of their own health status, including their past, present, and future health, as well as their resistance to disease and worries about health. Subjective health is considered a comprehensive indicator that integrates both objective health status and subjective mental health, and it has been widely used in domestic and international studies. The World Health Organization’s new definition of health (48) recognizes that health encompasses not only the absence of disease or physical fitness, but also physical and mental health, social well-being, and personal development. In this paper, subjective health (Shealth) and objective health (Ohealth) are included as separate indicators of residents’ health.

To measure subjective health, the study uses the question “What do you think of your current health status?” with response options ranging from “very unhealthy” to “very healthy,” with a corresponding value from 1 to 5. The higher the value, the healthier the individual is considered. To measure objective health, the study uses the question “Have you had any doctor’s diagnosis that requires hospitalization in the past year?” with a “Yes” answer indicating poor objective health (assigned a value of 0) and a “None” answer indicating good objective health (assigned a value of 1).

The core explanatory variable of this study is the urban digital economy. To measure the level of urban digital economy development, we adopt the approach of Tao et al. (9) that considers two dimensions: internet development and digital financial inclusion development. Internet development is measured by four indicators, namely internet penetration rate, related practitioners, related output, and cell phone penetration rate. The actual content of these indicators is reflected in the number of internet broadband access users per 100 people, the proportion of employees in the computer service and software industry relative to the proportion of employees in urban units, the total amount of telecommunications services per capital, and the number of cell phone users per 100 people. The original data for these indicators are sourced from the China Urban Statistical Yearbook. Meanwhile, for the development of digital financial inclusion, we use the China Digital Financial Inclusion Index (49), jointly compiled by the Digital Finance Research Center of Peking University and Ant Financial Services. In this study, the five tertiary indicators are processed through the entropy method to form the Urban Digital Economy Development Index (DEI). Furthermore, DEI2 represents the square of the digital economy development index. Table 1 displays the related evaluation index construction system.

**Table 1:** Evaluation index system of the development level of urban digital economy.

First class index	Second class index	Third class index	Attribute of indicator
Digital economy development indicators	Internet development level	Number of Internet broadband access users per 100 people	+
		Proportion of employees in computer services and software industry	+
		Total telecom services *per capita*	+
		Number of mobile phone users per 100 people	+
	Development level of digital inclusive finance	China Digital Inclusive Finance Index	+

On the mediating variables, this paper aims to explore environmental pollution as a mediating variable through which the development of the digital economy affects residents’ health. Specifically, we focus on four urban pollution indicators: industrial sulfur dioxide emissions, industrial nitrogen oxide emissions, industrial dust and soot emissions, and fine particulate matter concentration (PM2.5). These indicators were selected from the China Urban Statistical Yearbook and were processed by taking logarithms. Through using these pollution indicators, we aim to measure the pollution level of the residential environment and examine its potential mediating effect on the relationship between the digital economy and residents’ health.

In this study, given the scarcity of direct research on the relationship between the digital economy, environmental pollution, and residents’ health, control variables are primarily derived from articles investigating the impact of Internet use and environmental pollution on residents’ health. This study applies Grossman’s (6) health production function model as the theoretical framework and introduces pertinent variables that influence residents’ health. These variables encompass economic, medical, educational, social factors, and individual characteristics, serving as the primary control variables to mitigate potential confounding effects between the development of the digital economy and residents’ health outcomes. Additionally, the selection of control variables also informed by a comprehensive review of relevant literature, particularly the studies conducted by Li et al. (50) and Deng et al. (51). The chosen control variables are as follows.

(a) Economic and Medical Factors: Higher economic levels in an area tend to provide better health products, services, housing, and medical conditions, thus positively influencing health outcomes. In this paper, the logarithm of the gross domestic product (ln_gdp) of the city where the surveyed residents live is used to represent economic and medical factors. (b) Educational Factors: Education significantly impacts an individual’s quality of life, including employment opportunities, income level, nutritional access, adoption of healthy lifestyles, and medication efficiency. Therefore, in this paper, we use the number of years of education (eduy) of residents to measure the level of education of residents. (c) Social Factors: Urban areas generally have higher levels of science and technology, more efficient medical services, and better medical information. The urban–rural type of the resident’s place of residence (urban) is included in the control to account for differences in science and technology levels, urbanization development, and medical services between urban and rural areas. (d) Individual Characteristics: Controlling for individual characteristics is crucial in related studies. The controlled individual characteristics in this study include gender, age, perception of fairness, healthcare social insurance, smoking habits, alcohol consumption, physical condition (whether the person has been sick in the last 2 weeks), job status (whether the person has a job or not), and *per capita* income (income represented as logarithmic).

In the mechanism test, to enhance the robustness of our research conclusions, we further included city-level control variables in our analysis. These include: (a) city nighttime light data; (b) the logarithm of city population; (c) city terrain ruggedness; (d) distance of the city center to the nearest seaport; (e) city meteorological factors, including average annual temperature and average annual wind speed; (f) city traffic emissions, where we use total passenger (passenger) and freight (freight) traffic as proxy variables for transportation; (g) fossil fuel consumption, including total supply of man-made or natural gas (ng) and liquefied petroleum gas consumption (lpg). All these control variables could potentially affect the level of digital economy development or the degree of environmental pollution in cities, hence they are controlled to maintain the reliability of the research findings.

Descriptive statistics of all of the above variables are shown in Table 2.

**Table 2:** Descriptive statistics.

category	Variable	Observes	Mean	Std.Dev.	Min	Max
Interpreted variable	Shealth	53,904	3.633	0.992	1	5
	Ohealth	53,904	0.917	0.276	0	1
Core explanatory variables	DEI	53,904	0.107	0.119	0.009	0.868
	DEI2	53,904	0.0256	0.0750	8.37e-05	0.753
Control variable	ln_income	53,904	9.226	2.497	0	15.61
	Age	53,904	43.53	14.45	13	113
	Gender	53,904	0.476	0.499	0	1
	Eduy	53,904	8.950	3.857	1	23
	Justice	53,904	3.271	0.921	1	5
	Illness	53,904	0.120	0.324	0	1
	Job	53,904	0.637	0.481	0	1
	Insure	53,904	0.792	0.406	0	1
	Smoke	53,904	0.221	0.415	0	1
	Drink	53,904	0.173	0.378	0	1
	Urban	53,904	0.365	0.481	0	1
	ln_gdp	53,904	16.90	1.064	14.497	19.342
	Night lights	840	5.354	7.229	0	57.00
	lnpop(10000)	843	5.856	0.681	2.970	7.126
	Relief	843	0.700	0.815	0.00130	5.791
	lndistance(km)	843	5.482	1.477	1.020	7.931
	Temperature	836	11.17969	6.164722	−0.6477041	24.73545
	Wind speed	836	4.737797	1.046006	2.509084	8.642851
	Passenger (10000)	836	8111.74	14425.97	169	201,722
	Freight (10,000 tons)	836	10027.06	13437.65	441	272,423
	ng(10,000 m^3^)	836	22963.25	81786.98	1	2,099,057
	lpg(10,000 m^3^)	836	15289.42	33811.34	4	468,499
Intermediary variable	lnSo2	834	10.73	1.251	1.386	15.08
	lnNOx	785	10.02	1.137	5.403	15.00
	lnPM25	811	14.00	1.570	4.500	17.85
	lndirt	785	10.02	1.137	5.403	15.00

## 4 Empirical results and analysis

### 4.1 Benchmark regression results

Based on equations (1) and (2) to verify whether the development of the digital economy can affect the health of the residents, the results of the regression estimation are reported in Table 3. Column (1) shows that without adding control variables, the impact of digital economy on residents’ subjective health level decreases and then increases, where the primary term coefficient of digital economy is −1.034 and the secondary term coefficient is 1.025, and both are significant at the 1% level, indicating that there is a non-linear relationship between digital economy development and residents’ subjective health, and the relationship may show a U-shaped correlation. Column (2) shows that the primary term of the digital economy increases to −1.007 and the secondary term decreases to 0.972 after the inclusion of control variables. Column (3) shows that the impact of the digital economy on objective health without the inclusion of control variables also decreases and then increases, where the primary coefficient is −2.795, and the secondary coefficient is 2.481. The digital economy also has a non-linear impact on the objective health of residents, and there may also be a U-shaped relationship between the two. Column (4) shows that after adding the control variables, the primary coefficient is −2.710 and the secondary coefficient is 2.628, which are significant at the 5% level. Overall, the results suggest that there is a non-linear relationship between digital economy development and residents’ health, with the relationship possibly showing a U-shaped correlation.

**Table 3:** Benchmark regression results.

	(1)	(2)	(3)	(4)
	Shealth	Shealth	Ohealth	Ohealth
DEI	−1.034***	−1.007***	−2.795***	−2.710**
	(0.294)	(0.302)	(1.076)	(1.135)
DEI2	1.025***	0.972***	2.481*	2.628**
	(0.291)	(0.304)	(1.269)	(1.333)
ln_income		0.021***		0.022***
		(0.002)		(0.007)
Age		−0.026***		−0.018***
		(0.000)		(0.001)
Gender		0.119***		1.346***
		(0.011)		(0.057)
Eduy		0.022***		0.029***
		(0.002)		(0.006)
Justice		0.162***		0.108***
		(0.005)		(0.018)
Illness		−0.776***		−1.140***
		(0.015)		(0.042)
Job		0.183***		0.542***
		(0.010)		(0.037)
Insure		−0.038***		−0.263***
		(0.013)		(0.045)
Smoke		−0.098***		−2.528***
		(0.014)		(0.057)
Drink		0.062***		0.538***
		(0.014)		(0.050)
Urban		0.084***		0.044
		(0.015)		(0.055)
ln_gdp		0.181*		−0.708**
		(0.107)		(0.357)
City fixed effect	Yes	Yes	Yes	Yes
Year fixed effect	Yes	Yes	Yes	Yes
*N*	53,904	53,904	53,904	53,904

Note: Robust standard errors in brackets; ***, **, * significant at 1%, 5%, 10% respectively.

Scholars typically introduce the quadratic term of explanatory variables into the linear regression model to identify the “U”-shaped relationship between the explanatory variables and the explained variables. If the regression coefficient of the quadratic term is significant, it suggests a “U” or inverted U-shaped nonlinear relationship between the variables. However, Lind and Mehlum (47) argue that this criterion is no longer applicable if the results of the nonlinear fit are significantly concave and monotonic. Therefore, we refers to the three-step U-shaped test proposed by Lind and Mehlum (47) to ensure the robustness of the recognition of the “U-shaped” relationship. Equation (1) is simplified to $y=\beta_{1}X+\beta_{2}X^{2}+\beta_{3}$, and taking the derivative of the independent variable yields:$y^{\prime }=\beta_{1}+2\beta_{2}X$.Since the left and right endpoints of the core explanatory variables are 0.009 and 0.868, therefore, the slope at the endpoint can be obtained by substituting the X. And because when the slope $y^{\prime }$ = 0, the turning point can be found $X^{*}=-\beta_{1}$/$2\beta_{2}$, then we can determine whether the result is a U-shaped relationship.

The results of the U-shaped relationship test between the digital economy and the health of the residents are presented in Table 4. The results demonstrate that the coefficients of the primary and secondary terms of the regression estimates of the digital economy on the subjective and objective health of the residents have opposite properties of positive and negative. After adding control variables, the slopes of the left and right endpoints of the impact curve of the digital economy on residents’ subjective health were −0.990 and 0.680, respectively, and the slopes of the left and right endpoints of the impact curve of the digital economy on residents’ objective health were −2.663 and 1.852, respectively, indicating steep endpoint slopes. Additionally, the turning points of all four estimates fall within the range of values of the digital economy development index [0.009,0.868]. Therefore, it can be concluded that the U-shaped relationship between the digital economy and the subjective and objective health of the residents does exist. The findings suggest that in the short-term, an increase in the level of digital economy development is detrimental to the improvement of residents’ health, but in the long-term, the effect will gradually turn positive and show an increasing trend. Thus, in the long-term, the development of the digital economy will promote the improvement of residents’ health.

**Table 4:** Lind and Mehlum (47) U-test results.

	Shealth		Shealth		Ohealth		Ohealth	
$\beta_{1}$	−1.034		−1.007		−2.795		−2.710	
$\beta_{2}$	1.025		0.972		2.481		2.628	
Turning point	0.504		0.518		0.563		0.516	
Endpoint	Min	Max	Min	Max	Min	Max	Min	Max
Endpoint value	0.009	0.868	0.009	0.868	0.009	0.868	0.009	0.868
Kmin	−1.016		−0.990		−2.750		−2.663	
Kmax	0.745		0.680		1.512		1.852	
Shape	U-shape		U-shape		U-shape		U-shape	

Additionally, the results of the control variables estimation are generally consistent with existing literature. Firstly, at the individual level, individuals with higher education and personal income tend to have better health due to higher expenditure on health; men tend to be healthier than women (6); residents who perceive their social environment as fair tend to have better physical and mental health (52); employed residents tend to have higher health levels than unemployed residents, as work is related to income and mental health, which in turn affects health levels; older residents tend to have lower health levels, consistent with the depletion of health as a durable good over time (6); recent illness experiences significantly and substantially reduce health levels; smoking history tends to harm health, while alcohol consumption history can actually improve health status due to moderate alcohol consumption improving residents’ sense of self-satisfaction and promoting health. However, it is important to note that the survey data on alcohol consumption in the CLDS questionnaire is relatively crude, and the relationship between alcohol consumption and health may have endogeneity, requiring further examination. Additionally, residents with medical insurance tend to have lower health status, which may be due to adverse selection, where residents with lower health levels are more likely to buy medical insurance. Secondly, at the regional level, metropolitan areas and regions with higher levels of economic development tend to have more abundant medical resources and thus are more likely to benefit from better health outcomes.

### 4.2 Robustness test

To ensure the reliability of the study results, several robustness tests are conducted in this paper. The tests are as follows:

#### 4.2.1 Sample exclusion

The Yangtze River Delta region in China has the highest level of digital economy development, while other regions have much lower levels. Samples from the Yangtze River Delta are excluded to test the robustness of the study. Results presented in columns (1) and (2) of Table 5 show that even after excluding samples, the U-shaped relationship between digital economy development and residents’ subjective and objective health still holds.

**Table 5:** Robustness check.

	Remove some samples		Replacement sample		Control fixed effect	
	(1)	(2)	(3)	(4)	(5)	(6)
	Shealth	Ohealth	Shealth	Ohealth	Shealth	Ohealth
DEI	−0.853***	−2.612**	−2.762*	−4.529**	−0.997***	−2.646**
	(0.330)	(1.181)	(1.520)	(1.777)	(0.302)	(1.137)
DEI2	0.541*	2.525*	5.496*	10.347**	1.038***	2.447*
	(0.322)	(1.361)	(2.911)	(4.132)	(0.304)	(1.342)
Point of inflection	0.788	0.517	0.251	0.219	0.480	0.541
Kmin	−0.843	−2.567	−2.663	−4.343	−0.978	−2.602
Kmax	0.086	1.771	6.779	13.433	0.805	1.602
Shape	U-shape	U-shape	U-shape	U-shape	U-shape	U-shape
Control variable	Yes	Yes	Yes	Yes	Yes	Yes
Provincial effect	No	No	No	No	Yes	Yes
Province*Year	No	No	No	No	Yes	Yes
City fixed effect	Yes	Yes	Yes	Yes	Yes	Yes
Year fixed effect	Yes	Yes	No	No	Yes	Yes
*N*	47,937	48,181	10,993	11,002	53,904	53,904

Note: Robust standard errors in brackets; ***, **, * significant at 1%, 5%, 10% respectively.

#### 4.2.2 Replacement sample regression

To further test the robustness of the results, the sample is replaced with data from the 2018 CLDS survey. As shown in columns (3) and (4) of Table 5, the U-shaped relationship between digital economy development and residents’ subjective and objective health is still observed, indicating the robustness of the findings.

#### 4.2.3 Controlling for interaction fixed effects

Regions with a faster development and application of the digital economy tend to have higher levels of economic development, while more developed regions usually have better medical resources and health environments. This may lead to higher levels of population health, creating endogeneity problems for the causal relationship in the empirical analysis of this paper. To address this issue, the paper follows the approach of Zhao Tao et al. (9) and introduces province fixed effects and province-year interaction terms to mitigate the impact of macro-systemic environmental changes on residents’ health. Province fixed effects account for unobserved factors that are specific to each province and do not change over time, such as culture or geography. The province-year interaction term captures factors specific to each province that change over time, such as economic development or policy changes. By including these controls in the regression analysis, the impact of the digital economy on population health can be isolated from other potential confounding factors. Columns (5) and (6) of Table 5 demonstrate that the U-shaped relationship between digital economy development and population health remains significant after the inclusion of these interaction terms, confirming the robustness of the findings.

#### 4.2.4 Endogeneity test

In the regression concerning resident health mentioned above, we controlled for a number of micro-level individual variables, and the robustness tests have also adequately excluded possible measurement errors in the explanatory variables. Additionally, since the core explanatory variable, the digital economy index, is macro-level data, and the dependent variable, resident health, is micro-level individual data, there is no apparent reverse causality between them, which means that the endogeneity issue in this study is relatively minor. However, considering that there may still be problems with omitted variables and sample selection bias, to make the conclusions of the article more reliable and comprehensive, this paper uses the two-stage residual inclusion (2SRI) method to test for endogeneity in the model, in an effort to minimize the impact of endogeneity on the research conclusions.

Unlike previous studies, this paper faces the following two main problems in selecting instrumental variables. First, in this study, the impact of the digital economy on resident health is nonlinear, while traditional instrumental variable regression is usually only suitable for testing the endogeneity of linear relationships. Therefore, this paper needs to find instrumental variables for the square term of the digital economy. Second, the dependent variable in this study is a discrete choice variable, which means that the traditional two-stage least squares method cannot be used.

To address these issues, this paper adopts the two-stage residual inclusion (2SRI) method and chooses the number of post offices per million people and the number of landline telephones per hundred people in 1984 as instrumental variables. Postal and telecommunication services have historically been provided by the post and telecommunications departments, which laid the foundation for the later development of the digital economy, thereby satisfying the relevance condition. At the same time, the number of postal and telecommunication services in 1984, as historical data, has little impact on today’s resident health levels, thus meeting the exclusiveness restriction.

Since the historical number of postal and telecommunications services we used was cross-sectional data from 1984, it could not be directly applied to the analysis of mixed cross-sectional data. Therefore, this paper constructs an interaction term between the number of post offices per million people in 1984 and the number of international internet users in the year prior to the sample year as the instrumental variable for the first term of the digital economy. Simultaneously, we constructed the squared term of the interaction between the number of landline telephones per hundred people in 1984 and the number of international internet users from the year prior to the sample year as the instrumental variable for the second term of the digital economy. Using the 2SRI method, we first regress the endogenous explanatory variables using the instrumental variables in the first stage and extract the predicted values and residuals of the model. Then, in the second stage, we run the main Ordered Probit and Probit model regressions, but this time we include the predicted values and residuals from the first stage as explanatory variables in the regression. The regression coefficient of the predicted value is the impact of the digital economy on resident health after excluding endogeneity.

The 2SRI regression results, as shown in Table 6, are as follows: columns (1, 2) regress the endogenous variables DEI and DEI2 using the two instrumental variables, respectively, obtaining predicted values for each endogenous variable to exclude the exogenous part within the endogenous variables, that is, to attempt to explain the exogenous part of the variation in the explanatory variables, while also extracting residuals to reduce estimation bias. Columns (3, 4) include the predicted values and residuals to regress on resident health. We focus primarily on the predicted values of the digital economy and its second term. The results indicate that after excluding the interference of endogeneity, the first term of the digital economy on resident subjective health is negative, and the second term is positive, showing a U-shaped impact, and is significant at the 1% level, which means that endogeneity indeed affects the accuracy of regression estimation to some extent. Furthermore, the weak instrumental variable test result is 637.16, which is far greater than the critical value, proving the effectiveness of the instrumental variable.

**Table 6:** Endogeneity test.

	(1)	(2)	(3)	(4)
	DEI	DEI2	shealth	ohealth
IV	−0.000***	−0.000***		
	(0.000)	(0.000)		
IV2	0.000***	0.000***		
	(0.000)	(0.000)		
Predict of DEI			−1.616***	−2.549***
			(0.162)	(0.577)
Predict of DEI2			1.000***	3.389***
			(0.238)	(0.958)
Residuals of DEI			−1.646***	−3.195**
			(0.333)	(1.343)
Residuals of DEI2			1.255***	2.615*
			(0.419)	(1.581)
Point of inflection			0.808	0.376
Kmin			−1.598	−2.488
Kmax			0.120	3.334
Shape			U-shape	U-shape
Control variable	Yes	Yes	Yes	Yes
City fixed effect	Yes	Yes	Yes	Yes
Year fixed effect	Yes	Yes	Yes	Yes
Weak IV test	637.16			
*N*	39,367	39,367	39,367	39,367

Note: Robust standard errors in brackets; ***, **, * significant at 1%, 5%, 10% respectively.

## 5 Further discussion

### 5.1 Mechanism analysis

As literatures review noted, numerous studies in the literature have demonstrated that environmental pollution can have significant negative effects on the health of residents. Furthermore, the development of the digital economy has the potential to influence urban pollutant emissions and impact the health status of residents by altering the quality of the living environment. To effectively reveal the mechanism by which the digital economy affects the health of residents through changes in environmental quality, this paper initially constructs a spatial and temporal evolutionary pattern of digital economy and environmental pollution in order to explore the correlation between the evolutionary pattern of digital economy development level and changes in environmental pollution. The study then adopts the classical three-step approach proposed by Baron and Kenny (53) to test the path of the influence mechanism of digital economy on residents’ health. In step (1), the paper tests the influence of digital economic development on residents’ health, which has been previously demonstrated in the benchmark regression. In step (2), the paper tests the influence of digital economy development on the mediating variable of environmental pollution level. If the influence coefficient is significant, it indicates that the development of the digital economy can influence the level of environmental pollution. In step (3), the paper adds the mediating variable to the regression in step (1). If the regression coefficients of both the mediating variable and the core explanatory variable digital economy development index are significant, then it indicates that the mediating variable plays the role of partial mediation. Therefore, environmental pollution is established as a mechanism of digital economic development affecting residents’ health.

#### 5.1.1 Analysis of spatial and temporal evolution patterns of digital economy development and environmental pollution

To examine the spatial and temporal evolution characteristics of digital economy and environmental pollution at the urban scale in China, this study collected environmental pollution data from 296 cities and regions and combined it with urban digital economy index data. ArcGIS software was utilized to visualize and map the data.

**Figure 1:**
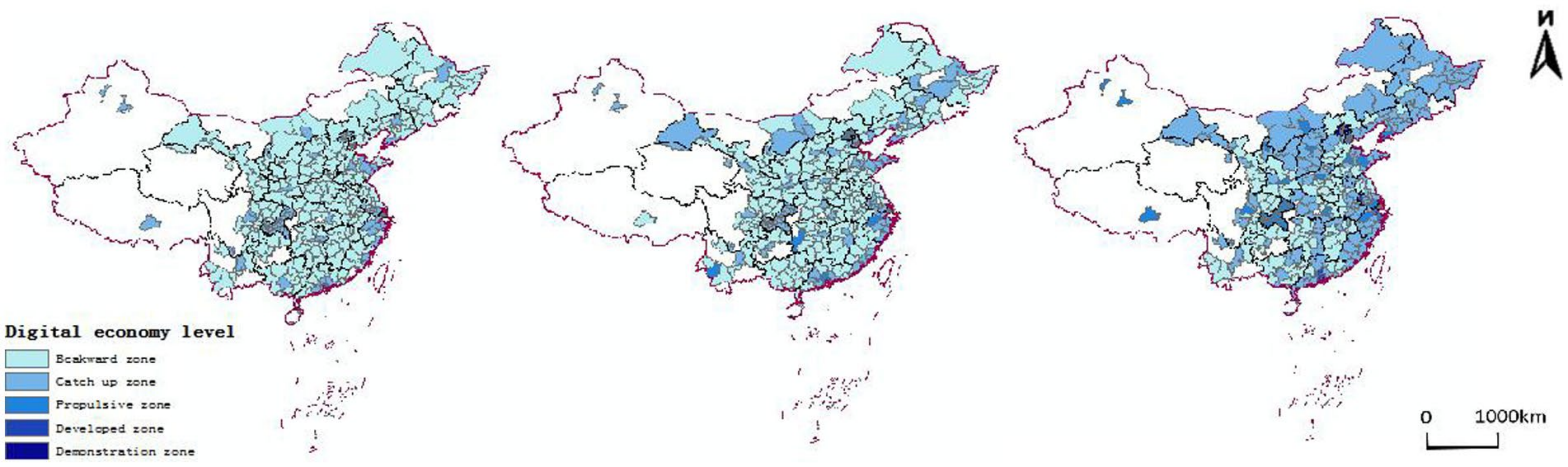
Spatial and temporal evolution pattern of China’s digital economy development level.

Distribution characteristics of digital economy development level evolution: The cities with digital economy development indexes in the range of [0–0.05], [0.05–0.15], [0.15–0.3], [0.3–0.5], and [0.5–1] were divided into five digital economy development levels, namely lagging area, catching up area, promoting area, developed area and demonstration areas. The space and temporal evolution pattern of the digital economy development level in 2011, 2013, and 2015 were drawn, as shown in Figure 1. As time progressed, the digital economy development pattern gradually evolved from a “multi-point” sporadic distribution with developed regions such as Beijing, Shanghai, Guangzhou, Shenzhen, Hangzhou, and Chongqing as the core to a “grouped and contiguous” distribution with the Yangtze River Delta Economic Zone, Pearl River Delta Economic Zone, Chengdu-Chongqing metropolitan area, and the Bohai Rim Economic Circle. Most cities have significantly developed their digital economy, especially in the northeast and northwest regions, and the overall development level of the digital economy has greatly improved.

**Figure 2:**
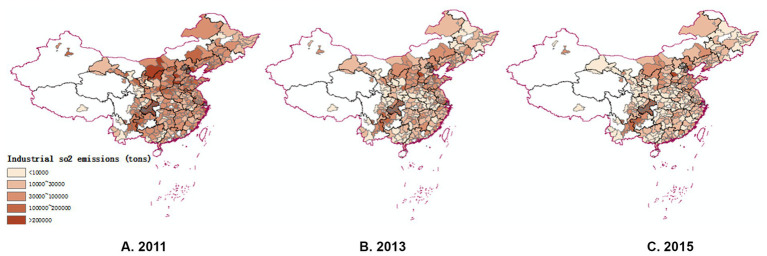
Spatial and temporal evolution pattern of China’s Industrial SO2 emissions level.

The evolution of the distribution characteristics of environmental pollution: To comprehensively examine the evolution of environmental pollution, this study selected industrial SO2 emissions, industrial nitrogen oxide emissions, industrial soot dust emissions, and fine particulate matter (PM2.5) as four indicators to measure environmental pollution in China. Due to spatial limitations, this paper focuses only on the spatial and temporal evolution of industrial sulfur dioxide emissions. The industrial SO2 emission data were divided into 5 levels based on the amount of emission: less than 10,000 tons, 10,000–30,000 tons, 30,000–100,000 tons, 100,000–200,000 tons, and more than 200,000 tons. The data were plotted using ArcGIS to obtain Figure 2. Generally, SO2 emission distribution is higher in the north and lower in the south, and higher in the west and lower in the east. This may be due to the heavier presence of the chemical industry in the north, which is a significant source of SO2. It can be observed that as time progresses, as the level of digital economy development significantly increases, there is a corresponding decrease in SO2 emissions. The effect of digital economy development in reducing pollution in the long term is initially tested through a comparative map of the spatial and temporal evolutionary patterns of the digital economy and industrial sulfur dioxide.

#### 5.1.2 Mechanism test

In order to verify the mechanism of the impact of the digital economy on the health of the residents through environmental pollution, the following mediating effect model is developed in this paper:


(4) $$M_{jt}=\lambda_{0}+\lambda_{1}DEI_{jt}+\lambda_{2}DEI2_{jt}+\lambda_{3}C_{M}+\sigma_{j}+\tau_{t}+\varepsilon_{jt}$$

(5) $$\begin{array}{l} Shealth_{ijt}=\mu_{0}+\mu_{1}DEI_{j,t-1}+\mu_{2}DEI2_{j,t-1}+\\ \;\;\;\;\;\;\;\;\;\;\;\;\;\;\;\;\;\;\;\;\;\;\;\;\;\;\;\;\mu_{3}M_{j,t-1}+\mu_{4}C+\sigma_{j}+\tau_{t}+\varepsilon_{ijt} \end{array}$$

(6) $$\begin{array}{l} Ohealth_{ijt}=\nu_{0}+\nu_{1}DEI_{j,t-1}+\nu_{2}DEI2_{j,t-1}+\\ \;\;\;\;\;\;\;\;\;\;\;\;\;\;\;\;\;\;\;\;\;\;\;\;\;\;\;\;\;\;\nu_{3}M_{j,t-1}+\nu_{4}C+\sigma_{j}+\tau_{t}+\varepsilon_{ijt} \end{array}$$

Where M is the mediating variable, representing four types of environmental pollution indicators, include industrial sulfur dioxide emissions (SO2), industrial nitrogen oxide emissions (NOx), industrial soot dust emissions (dirt) and fine particulate matter (PM2.5), $C_{M}$ represents the control variables in the second step of the mechanism test, including night lights, city population, city topography in terms of relief and the distance to nearest seaport, while the meanings of the other variables are the same as in equation (1).

The effects of digital economy on environmental pollution were tested by coefficients $\lambda_{1}$ and $\lambda_{2}$, the effects of digital economy on the subjective and objective health of the residents after controlling for the mediating variable M were tested by $\mu_{1}$ and $\mu_{2}$, $\nu_{1}$ and $\nu_{2}$, respectively, and the mechanisms of environmental pollution as a mediating variable on the health of the population were tested by $\mu_{3}$ with $\nu_{3}$.

Table 6 presents the results of the second step of the mediated effects test. It’s shows that the digital economy has an inverted U-shaped effect on industrial SO2, industrial NOx, PM2.5 as well as industrial soot and dust emissions, and the turning points are distributed around 0.2, with the continuous improvement of the level of urban digital economic development, pollutant emissions will show an inverted-U shape trend of first rising and then declining, which is exactly the opposite of the “U” shaped impact of the development of the digital economy on the health of the residents.

The possible reason is that the observed increasing trend of pollution emissions at the early stage of digital economy development in a region can be attributed to the immaturity of the digital economy. During the process of digital industrialization and industry digitalization, the high input and cost associated with the digital economy may result in higher levels of pollution emission in both production and living processes. This negative effect on environmental pollution can also negatively impact the health of residents. However, as the digital economy develops further, the initial costs and technology investments gradually have a positive effect, leading to improved energy use efficiency and optimized industrial structures, which can reduce pollutant emissions and create a healthier environment for residents. These findings regarding the inverted U-shaped influence relationship are consistent with previous studies such as Ahmadova et al. (27).

**Figure 3:**
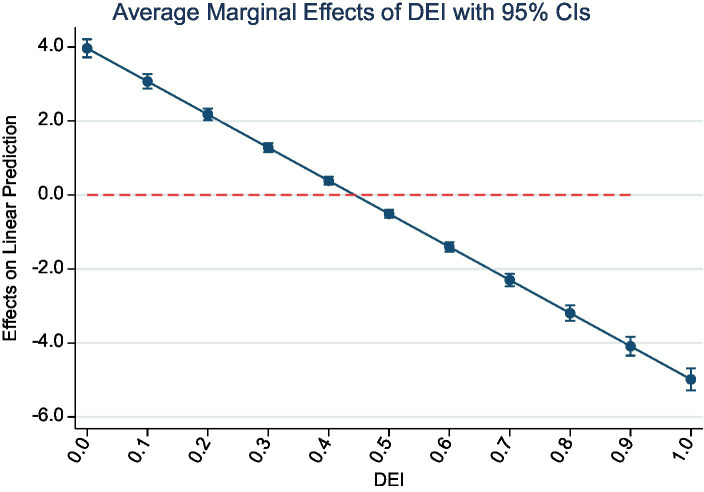
Marginal effect of DEI on SO2.

**Figure 4:**
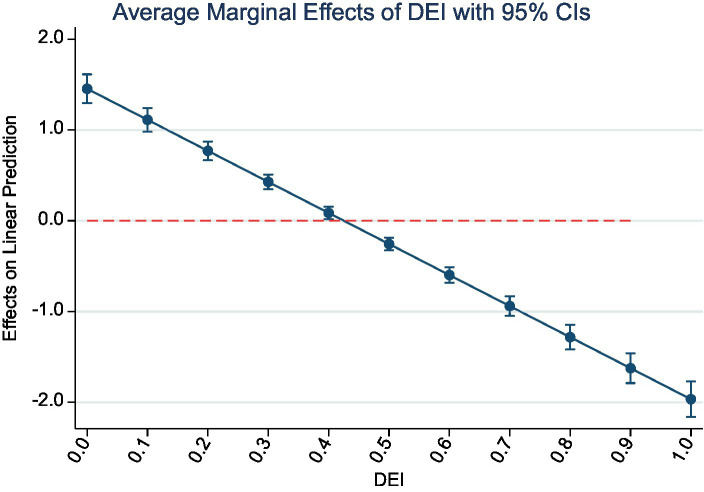
Marginal effect of DEI on dirt.

In order to further investigate the inverted U-shaped effect of the digital economy on environmental pollution, this study selected industrial sulfur dioxide and industrial dust and soot, which are highly hazardous to human health and can severely pollute the air environment and reduce air visibility, as examples. Marginal effects of digital economy development on pollutant emissions with 95% confidence intervals were plotted to analyze this relationship. Figures 3, 4 illustrate the marginal change in pollutant emissions caused by every 0.1 increase in the digital economy index, with the horizontal axis representing the urban digital economy index, and the vertical axis representing the marginal effect. The marginal effect of the digital economy index on the emission of both pollutants shows a sloping trend from the upper left to the lower right, and none of the confidence intervals cross 0, indicating a significant marginal effect of the digital economy index on pollution emission. The line above the red line represents that when the digital economy index is low, each 0.1 unit increase in the digital economy index causes an additional increase in industrial sulfur dioxide and industrial dust and soot emissions, while the line below the red line indicates that when the digital economy continues to develop until the index is greater than 0.5, each 0.1 unit increase in the digital economy development index results in an additional reduction in the emissions of the two pollutants. This result is consistent with the findings of the second step regression of the intermediary test above.

After examining the inverted U-shaped effect of digital economy development on environmental pollution, the paper proceeds to examine the third step of the mediating effect. Table 7 reports the regression of digital economy on residents’ health after controlling for each of the four types of environmental pollutants. After controlling for the four types of pollutants, the digital economy still maintains a significant U-shaped effect on both the subjective and objective health of the residents. Columns (1) and (2) show that SO2 has a significant negative effect on residents’ objective health, but not on subjective health. Industrial sulfur dioxide plays a partially mediating role on residents’ objective health. Columns (3) and (4) show that industrial dust and smoke has a significant negative effect on both residents’ subjective and objective health. Industrial dust and smoke plays a partially mediating role on both residents’ subjective and objective health. Columns (5) and (6) show that industrial nitrogen oxides do not have a significant effect on residents’ subjective and objective health. Columns (7) and (8) show that PM2.5 has a significant negative effect on residents’ subjective health, but not on objective health. PM2.5 has a partially mediating effect on residents’ subjective health.

**Table 7:** Digital economy development level and Environmental Pollution.

	(1)	(2)	(3)	(4)
	lnSo2	lnNOx	PM2.5	lndirt
DEI	5.216**	6.265***	3.778*	6.265***
	(2.293)	(1.794)	(2.287)	(1.794)
DEI2	−13.948**	−12.509***	−9.177***	−12.509***
	(5.629)	(3.274)	(2.946)	(3.274)
Light	0.025***	0.014**	0.028***	0.014**
	(0.009)	(0.006)	(0.011)	(0.006)
lnpop	0.392***	0.503***	0.469***	0.503***
	(0.082)	(0.069)	(0.098)	(0.069)
Relief	0.068	0.056	−0.166	0.056
	(0.080)	(0.060)	(0.120)	(0.060)
Distance	0.043	0.015	0.080*	0.015
	(0.031)	(0.037)	(0.046)	(0.037)
Passenger	−0.000	−0.000**	−0.000	−0.000**
	(0.000)	(0.000)	(0.000)	(0.000)
Freight	0.000	0.000	0.000	0.000
	(0.000)	(0.000)	(0.000)	(0.000)
Temp	−0.014	−0.074***	−0.065***	−0.074***
	(0.013)	(0.010)	(0.015)	(0.010)
Wind	0.054	0.084*	0.132**	0.084*
	(0.047)	(0.043)	(0.063)	(0.043)
Naturegas	−0.000	−0.000	0.000	−0.000
	(0.000)	(0.000)	(0.000)	(0.000)
lpg	−0.000	−0.000***	−0.000	−0.000***
	(0.000)	(0.000)	(0.000)	(0.000)
Turning point	0.187	0.250	0.206	0.250
Kmin	4.965	6.040	3.613	6.040
Kmax	−18.998	−15.451	−12.153	−15.451
Shape	Inverted U-shaped	Inverted U-shaped	Inverted U-shaped	Inverted U-shaped
Year FE	Yes	Yes	Yes	Yes
*N*	824	775	801	775
adj. *R*^2^	0.239	0.227	0.120	0.227

Note: Robust standard errors in brackets; ***, **, * significant at 1%, 5%, 10% respectively.

In general, the coefficients of the primary term of the digital economy on residents’ subjective and objective health are negative, and those of the secondary term are positive. After adding intermediary variables, when the coefficient of the intermediary variable is significant, the absolute values of the first and second coefficients of the digital economy on residents’ health decrease. This indicates that environmental pollution has played a partial mediating role. That is, the digital economy indirectly has a U-shaped effect on residents’ health by exerting an inverted U-shaped effect on environmental pollution. Additionally, the three categories of environmental pollution, namely industrial sulfur dioxide, industrial dust and smoke, and PM2.5, have a suppressive effect on residents’ health level, and the health condition of the residents will gradually improve as the pollution level decreases.

Furthermore, with regards to the turning point of the digital economy on residents’ health, it remains at 0.5 even after accounting for the four pollutant mediating variables. This indicates that when the index of digital economy development in cities exceeds 0.5, the digital economy will gradually promote the health of residents. However, the average level of digital economy in Chinese cities is only about 0.1, which is much smaller than the turning point of 0.5 Only cities such as Beijing, Shenzhen, Hangzhou, and Shanghai have surpassed the turning point and exerted a positive effect of digital economy on health. This suggests that the digital economy in China is mainly distributed on the left side of the U-shape curve, and its effect on residents’ health is primarily inhibitory. Additionally, the turning point of the digital economy on the level of environmental pollution is above 0.35 for all pollutants, which is also higher than the average level. This indicates that the pollution reduction effect of the digital economy is still in a relatively early stage, and the positive effect on pollution reduction needs to be further stimulated.

The analysis of the impact mechanism has confirmed that the level of environmental pollutant emissions plays a significant mediating role in the impact of the digital economy on residents’ health. Moreover, the impact of the digital economy on pollutant emissions follows an inverted U-shape, while its impact on health has a U-shape. These findings suggest that the mechanism of environmental pollution as a part of the mediating passed the test. In the long run, the sustainable development of urban digital economy is expected to reduce the level of environmental pollution and improve the subjective and objective health of residents by improving environmental quality.

### 5.2 Heterogeneity analysis

The development of China’s digital economy exhibits significant disparities between urban and rural areas, and the health status of residents differs considerably among various age groups. Therefore, it is imperative to explore in more detail the heterogeneity of the impact of digital economy development on residents’ health across different regions and age brackets.

#### 5.2.1 Distinguish between urban and rural areas

At present, China exhibits a significant urban–rural dichotomy, with a substantial digital divide existing between urban and rural residents, and digital inequality in certain regions is even more pronounced than economic inequality (54). Hence, exploring the heterogeneous effects of the digital economy on the health of these residents holds practical significance.

The sample at the urban–rural level was categorized based on the area where individuals were surveyed. The study investigated the varied effects of digital economy development on the health levels of diverse urban and rural residents through group regressions, with the findings reported in columns (1)–(4) of Table 8. The study reveals that the digital economy has significant effects on the subjective and objective health of residents in rural areas, while none of the effects on the health of urban residents are significant, demonstrating urban–rural heterogeneity. The primary and secondary coefficients of the digital economy on the subjective health of rural residents are −2.894 and 3.619, respectively, whereas the primary and secondary coefficients on the objective health of rural residents are −5.488 and 6.139, respectively. The turning point of subjective health is 0.400, and the turning point of objective health is 0.447, which are lower than the turning point of the full-sample benchmark regression. These findings suggest that the digital economy will promote the health level, especially the subjective health level, of rural residents at a faster pace.

**Table 8:** Digital economy, environmental pollution and residents’ health.

	(1)	(2)	(3)	(4)	(5)	(6)	(7)	(8)
	Shealth	Ohealth	Shealth	Ohealth	Shealth	Ohealth	Shealth	Ohealth
DEI	−1.036***	−2.444**	−0.949***	−2.562**	−1.004***	−2.642**	−0.979***	−2.734**
	(0.306)	(1.144)	(0.303)	(1.140)	(0.302)	(1.140)	(0.302)	(1.134)
DEI2	1.005***	2.382*	0.905***	2.511*	0.973***	2.566*	0.954***	2.780**
	(0.308)	(1.343)	(0.305)	(1.335)	(0.305)	(1.339)	(0.303)	(1.332)
lnso2	0.010	−0.074**						
	(0.011)	(0.036)						
lndirt			−0.032**	−0.101*				
			(0.016)	(0.052)				
lnNOx					0.005	−0.058		
					(0.012)	(0.042)		
lnPM25							−0.126**	−0.319
							(0.064)	(0.217)
Turning point	0.515	0.513	0.524	0.510	0.516	0.515	0.513	0.492
Kmin	−1.018	−2.401	−0.933	−2.517	−0.986	−2.596	−0.962	−2.684
Kmax	0.709	1.691	0.622	1.797	0.685	1.813	0.677	2.092
Shape	U-shape	U-shape	U-shape	U-shape	U-shape	U-shape	U-shape	U-shape
Control	Yes	Yes	Yes	Yes	Yes	Yes	Yes	Yes
FE	Yes	Yes	Yes	Yes	Yes	Yes	Yes	Yes
*N*	53,904	53,801	53,904	53,801	53,904	53,801	53,904	53,801

Note: Robust standard errors in brackets; ***, **, * significant at 1%, 5%, 10% respectively.

#### 5.2.2 Distinguish the age of the residents

With the sharp decline in the fertility rate in China in recent years, the issue of aging has become an important social concern. It is equally important to explore the impact of the digital economy on the health of the older adult.

In this study, the sample was divided into two groups based on the age of the questionnaire respondents: the older adult group aged 60 and above and the middle-aged and young group below 60. Group regressions were conducted, and the results were organized into columns (5)–(8) of Table 8. The regression results indicate that the digital economy has a significant effect on the subjective health of both the older adult group and the young and middle-aged group, while the effect on objective health is not significant, and there is age heterogeneity. Among the older adult residents aged 60 years or older, the primary term coefficient of the digital economy on subjective health was −2.114, and the secondary term coefficient was 2.355, while the effect on objective health was not significant. Among the young and middle-aged residents aged less than 60 years, the primary term coefficient of the digital economy’s impact on subjective health was −0.826, and the secondary term was 0.813, while the effect on objective health was also not significant. The results of the U-shaped test indicate a U-shaped effect of the digital economy on the subjective health of the older adult and the middle-aged and young people. The turning point of the “U” curve for the subjective health of the older adult is 0.449, which is earlier than the turning point of 0.508 for the subjective health of the middle-aged and young people, indicating that the digital economy will promote the health level of the older adult more quickly.

The results of the above heterogeneity analysis indicate that the impact of digital economy development on the health of the residents varies by area of residence and age group. The digital economy has a more significant impact on the health level of rural areas and older adult groups compared to those in urban areas and older adult groups, and it reaches the rising turning point earlier (Table 9).

**Table 9:** Heterogeneity analysis.

	(1)	(2)	(3)	(4)	(5)	(6)	(7)	(8)
	Country Shealth	Urban Shealth	Country Ohealth	Urban Ohealth	Age < 60 Shealth	Age ≥ 60 Shealth	Age < 60 Ohealth	Age ≥ 60 Ohealth
DEI	−2.894***	0.105	−5.488***	−0.531	−0.826**	−2.114**	−2.187	−4.037
	(0.534)	(0.418)	(1.959)	(1.672)	(0.323)	(0.907)	(1.331)	(2.750)
DEI2	3.619***	−0.069	6.139**	0.431	0.813**	2.355**	2.120	5.075
	(0.814)	(0.386)	(3.021)	(1.687)	(0.325)	(1.145)	(1.500)	(3.833)
Point of inflection	0.400	0.761	0.447	0.616	0.508	0.449	0.516	0.398
Kmin	−2.829	0.104	−5.377	−0.523	−0.811	−2.072	−2.149	−3.946
Kmax	3.389	−0.015	5.169	0.217	0.585	1.974	1.493	4.773
Shape	U-shape		U-shape		U-shape	U-shape		
Control variable	Yes	Yes	Yes	Yes	Yes	Yes	Yes	Yes
City FE	Yes	Yes	Yes	Yes	Yes	Yes	Yes	Yes
Year FE	Yes	Yes	Yes	Yes	Yes	Yes	Yes	Yes
*N*	34,225	19,679	34,179	19,558	46,051	7,850	45,927	7,710

Note: Robust standard errors in brackets; ***, **, * significant at 1%, 5%, 10% respectively.

## 6 Conclusion and discussion

### 6.1 Research findings

**Figure 5:**
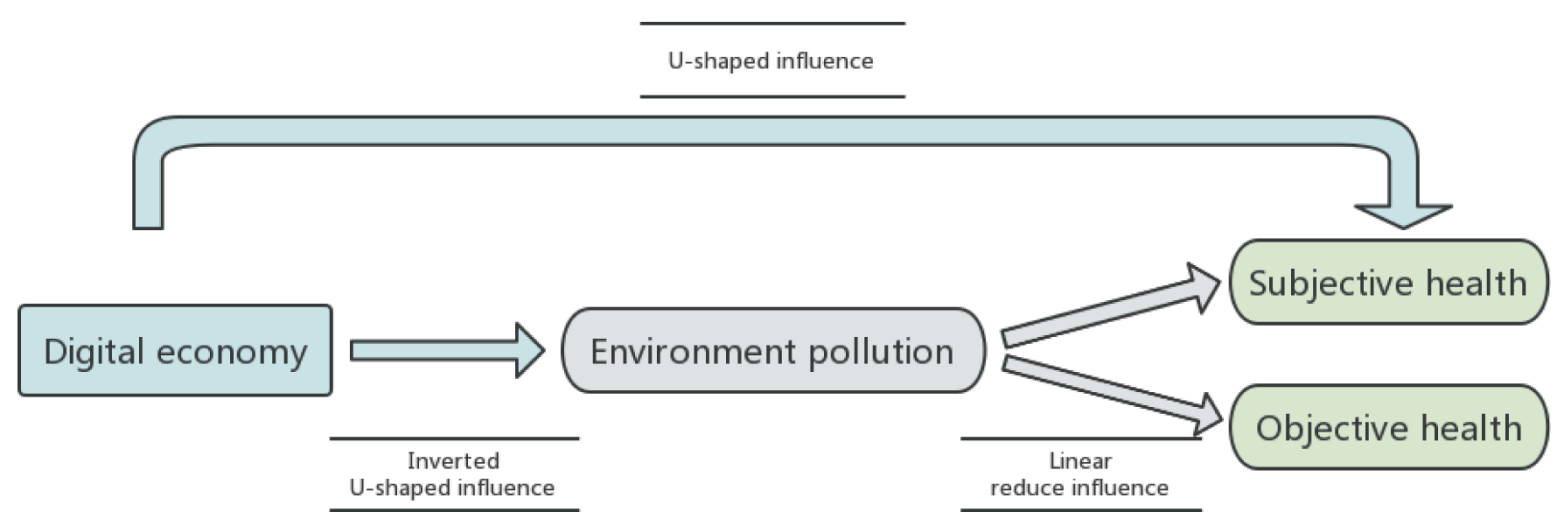
Research conclusion.

In the context of building digital China and health China strategy, this paper explores the impact of the digital economy on the subjective and objective health of Chinese residents, as well as the impact mechanism effect of environmental pollution. With the understanding that the development of the digital economy will significantly transform the quality of China’s economic development and reshape the lifestyles of individuals, this study analyzes mixed cross-sectional data from CLDS in 2012, 2014, and 2016. The study investigates the development of the digital economy and its impact on residents’ health, as well as the role of environmental pollution in this relationship. The research conclusion of this article can be visually presented through the following Figure 5.

Firstly, the digital economy exhibits a pronounced U-shaped impact on residents’ subjective and objective health. As the digital economy progresses, residents’ health initially declines before improving. The “U” curve’s turning points for subjective and objective health are 0.518 and 0.516, respectively, substantially exceeding the digital economy’s average level of 1.07. Most regions currently lie on the left side of this curve, indicating that, during this period, the digital economy predominantly exerts an inhibitory effect on residents’ health. These conclusions are corroborated by robustness tests, which include sample replacement, exclusion of the Yangtze River Delta sample, and consideration of interaction fixed effects.

Secondly, the study demonstrates that the digital economy significantly impacts residents’ health through environmental pollution. The research uncovers an inverted U-shaped relationship between the digital economy’s effect on industrial SO2, NOx, PM2.5, and industrial soot and dust emissions, initially increasing and then decreasing. This pattern contrasts with the U-shaped influence of the digital economy on residents’ health. The environmental pollutants’ turning points are around 0.2, and the findings suggest that environmental pollution mediates the digital economy’s effect on health.

Thirdly, the digital economy’s impact on health demonstrates considerable urban–rural and age-related heterogeneity. The analysis indicates a significant U-shaped effect of the digital economy on rural residents’ health, but not on urban residents. This points to urban–rural disparities. Additionally, the digital economy affects the subjective health of individuals aged 60 and above, as well as those under 60, in a U-shaped manner. However, the turning point for the older adults subjective health impact is lower, and the objective health impact is not significant, highlighting age-related differences in the digital economy’s health effects.

### 6.2 Policy recommendations

This study not only furnishes empirical evidence regarding the digital economy’s impact on residents’ health but also offers key policy recommendations as follow.

Firstly, a global emphasis on continually advancing and strengthening digital economy infrastructure is paramount. In numerous cities, including those in China, the digital economy is still nascent, a phase during which it might temporarily impair resident health and intensify environmental pollution. To optimize the digital economy’s benefits and alleviate its early negative effects, global policies should endorse the expansion of telecommunications networks, 5G base stations, and similar infrastructures. This initiative should encompass increased government funding, tax incentives, and support for technological innovation. Long-term planning for digital infrastructure must include the implementation of high-speed broadband, extensive 5G network coverage, and cloud computing services, particularly in areas lagging in digital development. Governments should offer financial subsidies, low-interest loans, and tax breaks to incentivize private investment, and establish comprehensive regulatory frameworks for the digital economy to ensure fair competition and data security.

Second, the development of the digital economy should be actively promoted to fully utilize its potential in environmental protection, energy conservation, and emission reduction. Governments should continue to promote industrial digitalization and the digitalization of industries, building a new economic model centered on the digital economy. The focus should be on applying digital technology in production process optimization, emission reduction technology, energy management, precision agriculture, and popularizing it in living areas such as smart homes and online services. Additionally, select some cities or regions as pilot projects for combining digital economy with environmental protection, to demonstrate their effectiveness.

Thirdly, to bridge the global digital divide, extending the digital economy’s reach is essential. This entails boosting infrastructure investments to provide high-quality network services in rural and digitally underdeveloped regions. Concurrently, global digital skill enhancement programs, particularly for the older adult and rural populace, are vital. Digitizing government services is also crucial, as it simplifies access to health, education, and social security information for residents and improves user experience, ensuring inclusive benefits from digital transformation.

### 6.3 Limitation

This paper studies the impact of the digital economy on the health of residents but acknowledges certain limitations. Firstly, due to data constraints, it focuses on the effects of China’s digital economy development prior to 2018. With the further advancement of the digital economy over the past 5 years, the trends and impacts of China’s digital economy may have changed. Secondly, the paper only discusses the mechanisms of environmental pollution, overlooking other equally important mechanisms and regulatory roles such as the application of digital information technology, the digitization of public health services, and the healthcare system. Finally, the paper primarily examines the impact of the digital economy on individual health based on microdata, without evaluating its broader implications on the health of the population from a macro perspective, such as regional birth rates, mortality rates, and the incidence of cardio-pulmonary diseases. Therefore, in future research, we will continue to explore the impact of the digital economy on the physical and mental health of residents at the macro level.

## Data availability statement

The original contributions presented in the study are included in the article/supplementary material, further inquiries can be directed to the corresponding author.

## Author contributions

CZ: responsible for selecting paper topics, measuring indicators, and proofreading. ZW: responsible for writing papers and processing data. BS: responsible for controlling the direction of the paper and providing guidance. YY: responsible for handling the endogeneity of the empirical part of the article and revising the content of the article. All authors contributed to the article and approved the submitted version.
